# Novel *COL2A1* variants in Japanese patients with spondyloepiphyseal dysplasia congenita

**DOI:** 10.1038/s41439-022-00193-x

**Published:** 2022-05-17

**Authors:** Moe Akahira-Azuma, Yumi Enomoto, Naoyuki Nakamura, Takayuki Yokoi, Mari Minatogawa, Noriaki Harada, Yoshinori Tsurusaki, Kenji Kurosawa

**Affiliations:** 1 Division of Medical Genetics, Kanagawa Children’s Medical Center, Yokohama, Japan; 2 Clinical Research Institute, Kanagawa Children’s Medical Center, Yokohama, Japan; 3 Department of Orthopedic Surgery, Kanagawa Children’s Medical Center, Yokohama, Japan; 4 Department of Clinical Laboratory, Kanagawa Children’s Medical Center, Yokohama, Japan

**Keywords:** Disease genetics, Genetic testing

## Abstract

Spondyloepiphyseal dysplasia congenita (SEDC) is a multisystemic skeletal disorder caused by pathogenic variants in *COL2A1*. Here, we report the genotype-phenotype correlations in five Japanese patients with SEDC based on their clinical and radiological findings. All five patients had novel missense variants resulting in glycine substitutions (G474V, G543E, G567S, G594R, and G1170R). Genetic testing is important for early intervention for the extraskeletal complications of SEDC. Spondyloepiphyseal dysplasia congenita (SEDC) (OMIM#183900) is an autosomal dominant chondrodysplasia characterized by disproportionate short stature, abnormal epiphyses, flattened vertebral bodies (skeletal abnormalities), and extraskeletal features, including myopia, retinal degeneration with retinal detachment, and cleft palate. SEDC is caused by a heterozygous variant in the collagen II alpha 1 (*COL2A1*) gene.

Type II collagen diseases, collectively termed type II collagenopathies, show variable phenotypes. These pathologies included SEDC, Stickler syndrome (OMIM #108300), Kniest dysplasia (OMIM #156550), spondyloperipheral dysplasia (OMIM #271700), SED with metatarsal shortening (OMIM #609162), achondrogenesis type 2 (OMIM #200610), and platyspondylic dysplasia Torrance type (OMIM #151210). More than 200 variants have been reported in type II collagenopathies to date. Clarifying the genotype-phenotype correlation and nonskeletal features of these disorders is important for medical management and genetic counseling^1–3^. SEDC, in particular, has a wide range of clinical manifestations, and new causative variants continue to be reported^4–6^. Here, we describe the phenotypes of five Japanese patients with SEDC who carry a *COL2A1* pathogenic variant and have a wide range of medical problems. We investigated the genotype-phenotype correlation and compared our findings with those previously reported for SEDC.

We retrospectively reviewed the medical records of five children radiologically diagnosed with SEDC who visited the Genetics Clinic at Kanagawa Children’s Medical Center (KCMC) between April 2016 and March 2018. The clinical information examined included personal medical history, family history, skeletal surveys, physical examination findings and orthopedic, ophthalmologic, audiologic, and otolaryngologic problems.

Written informed consent was obtained from the parents, in accordance with the KCMC Review Board and Ethics Committee. Genomic DNA was extracted from peripheral blood samples. Extracted DNA was sequenced using the TruSight One Sequencing Panel on a MiSeq platform (Illumina, Inc., San Diego, CA) with 151-bp paired-end reads. Exome data alignment, variant calling, and variant annotation were performed as previously described^7,8^. The average coverage depth in the targeted regions exceeded 45 reads, and more than 95% of the targeted bases had coverage above 10x.

Table 1 summarizes the clinical manifestations and variants identified. All five patients had novel missense mutations resulting in glycine substitutions (G474V, G543E, G567S, G594R, and G1170R). All the variants are absent in GnomAD^9^ and are classified as likely pathogenic according to ACMG criteria^10^. Recently, the G594R and G1170R variants were evaluated as pathogenic and likely pathogenic in ClinVar (laboratory submission), respectively. However, detailed clinical manifestations were not described. The variants were de novo in three patients and inherited from their parents in the remaining two (Patient 2 inherited it from his mother and Patient 4 inherited it from her father). Four of the patients were born at term (37–39 weeks), and one was born preterm (35 weeks). All five patients were short for their gestational age at birth (−1.2 to −3.2 SD), and growth failure (−1.5 to −6.3 SD) continued due to skeletal dysplasia. The five patients had arthropathies associated with coxa vara, coxa valgus, and Perthes-like deformities. Vertebral involvement included cervical vertebral plana and odontoid hypoplasia. Two patients had ophthalmologic problems, and one had hearing problems. In addition to short stature and orthopedic problems, Patient 1 had cleft palate and micrognathia, which are common in Stickler syndrome. Patient 2 showed amblyopia and hearing loss. Patient 3 had ophthalmologic findings, such as hyperopia and astigmatism. Patients 4 and 5 had no ophthalmologic or audiological findings.

**Table 1:** Clinical manifestations and *COL2A1* variants of five Japanese children with spondyloepiphyseal dysplasia congenita.

Individual patient	1	2	3	4	5
Gene mutation (COL2A1:NM_001844.5)	exon23:c.1421G>T:p.G474V (novel)	exon25:c.1628G>A:p.G543E (novel)	exon26:c.1699G>A:p.G567S (novel)	exon27:c.1780G>A:p.G594R (ClinVar: pathogenic)	exon50:c.3508G>C:p.G1170R (ClinVar: likely pathogenic)
Inheritance	De novo	Maternal inheritance	De novo	Paternal inheritance	De novo
Age at genetic testing	11 months	7 years 3 months	2 years 4 months	7 years 11 months	2 years 5 months
Sex	Female	Male	Female	Female	Male
Prenatal growth					
Gestation	39 weeks 2 days	35 weeks 0 days	39 weeks 3 days	39 weeks 0 days	39 weeks 4 days
Birth length (cm)	45 (−2.1 SD)	36 (−3.2 SD)	45 (−2.1 SD)	46.2 (−1.3 SD)	46.0 (−1.2 SD)
Birth weight (g)	2650 (−1.1 SD)	2074 (−0.82 SD)	3176 (−0.44 SD)	2768 (−0.30 SD)	2642 (−1.2 SD)
Head circumference (cm)	32.5 (−0.56 SD)	N/A	32.0 (−0.97 SD)	33 (−0.16 SD)	33.9 (−0.46 SD)
Postnatal growth					
Age when measured	N/A	7 years 2 months	4 years 1 month	8 years 11 months	2 years 4 months
Height (cm)	N/A	71.5 (−9.6 SD)	86.5 (−3.6 SD)	121.0 (−1.5 SD)	68.2 (−6.3 SD)
Weight (kg)	N/A	11.1 (−3.0 SD)	13.5 (−0.9 SD)	22.6 (−1.0 SD)	8.45 (−3.0 SD)
Head circumference (cm)	N/A	N/A	44.2 (−0.3 SD)	51.0 (+0.5 SD)	49 (+0.1 SD)
Orthopedic findings	N/A	Bilateral femoral bone exodeviation and no cervical spinal stenosis at 3 years 6 months, no visaible femoral bone epiphysis at 9 years	Cervical vertebra plana, odontoid hypoplasia	Mild bilateral pes planovalgus at 1 year 9 months, Perthes-like deformity at 3 years	Coxa vara, flat feet, cervical cord compression due to C1-C2 instability
Ophthalmological findings	Refer	Amblyopia (eye glasses)	Hyperopia, astigmatism	No abonormality	Refer
Audiological findings	N/A	Sensorineuroal hearing loss (hearing aids)	Normal	No abonormality	N/A
Oropharyngeal findings	Cleft palated, micrognathia				N/A
Physical examination findings	Short stature	Short stature	Short extremities, short neck	Short stature	Short stature, relative macrocephaly
Developmental milestones	N/A	Head control 2 years 6 months, roll over 2 years 9 months, crawl 3 years, unable to stand with support at 9 years	Head control 4 months, roll over 5 months, crawl 6 months, stand with support 8 months, walk with support 11 months	Head control 3 months, sit unsupported 9 months, stand with support 10 months, stand unsupported 12 months, walk unsupported 15 months	Walk unsupported 2 years 4 months, speech normal
Other findings		Chronic lung failure, tracheostomy, oxygen requirement			

Figure 1B shows the growth charts for Patients 2, 3, and 5. Five patients had disproportionate short stature with varying severity (mild in Patient 4 to severe in Patient 1). Patient 2’s skeletal survey showed significant platyspondyly, shortening of long bones with ragged metaphyses, mild iliac hypoplasia, and delayed ossification of the femur head (Fig. 1C).

**Fig. 1:**
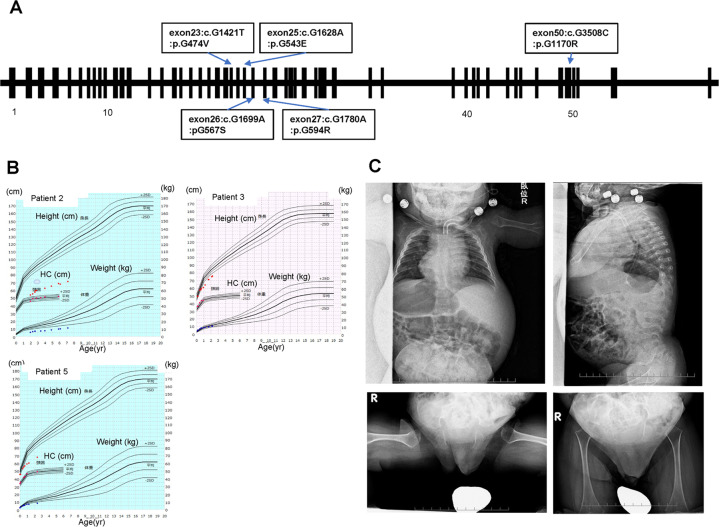
Clinical and genetic findings of five children with spondyloepiphyseal dysplasia congenita. **A** Distribution of the six novel *COL2A1* variants that were identified. **B** Growth charts (Japanese version 2020) for Patients 2, 3, and 5. **C** Skeletal survey for Patient 2. Radiographic findings included significant platyspondyly, shortening of long bones with ragged metaphyses, mild iliac hypoplasia, and delayed ossification of the femur head.

We confirmed the diagnosis based on the identification of *COL2A1* pathogenic variants by NGS and Sanger sequencing. Two cases were familial. The mother of Patient 2 was also affected by SEDC. Her first child was a normally developing female, and her second child was Patient 2. Both were delivered by cesarean section. The mother of Patient 2’s height was 112 cm (−8.5 SD). She had been under medical management for astigmatism, amblyopia, submucosal cleft palate, and coxa vara. The father of Patient 4 had been treated for hip dislocation in childhood and was clinically diagnosed with multiple epiphyseal dysplasia. His height was 151 cm (−3.4 SD). He had been under management for scoliosis, myopia, and osteoarthritis. These results suggest that intrafamilial phenotypic variation occurs in SEDC and that molecular testing can play an important role in making a definitive diagnosis.

Many glycine substitutions have been previously reported^1–3^. Overall, glycine to serine substitutions shows highly variable phenotypes, from milder to more severe forms. However, glycine to nonserine residue substitutions exclusively result in mild phenotypes. Four variants from our patients—G474V, G543E, G567S, and G594R—were located in the triple-helical domain of the procollagen alpha 1(II) chain. Patient 3 (p.G567S) showed a less severe form with a glycine to serine substitution. In line with previous reports, these observations may support the notion of residue-specific phenotype or severity in patients with SEDC.

Patient 5 (p.G1170R) showed a typical phenotype of SEDC, with severe coxa vara, short stature, and cervical cord compression due to C1-C2 instability. Notably, at the same codon position, p.G1170S is a recurrent variant that has been shown to cause avascular necrosis of the femoral head (ANFH) or Legg-Calve-Perthes disease (LCPD) but without the cardinal features of SEDC. All of the patients reported thus far are from East Asia^11–13^. Patient 5 is now 5 years old and is able to walk but has hip pain. As he grows, careful follow-up is needed to see if he develops ANFH or LCPD, as in the case of individuals with the p.G1170S variant.

Terhal et al. reviewed the clinical and radiological features in a cohort of 93 patients with SEDC or related disorders due to *COL2A1* variants and proposed guidelines for the management and follow-up of type II collagenopathies^2^. They reported that 86% of patients had short stature, more than 50% underwent orthopedic surgery, 45% had myopia and 37% had hearing loss as nonskeletal complications. Our five patients also presented with variable nonskeletal complications, suggesting the importance of genetic diagnosis for early intervention and prevention. However, it is difficult to establish clear rules for a genotype-phenotype correlation in SEDC. Other genetic or nongenetic factors could be involved that have not yet been discovered.

In conclusion, genetic testing can provide definitive diagnosis in patients with SEDC. Since the different manifestations may involve not only domain-specific but also codon-specific underlying mechanisms, it would be extremely useful to accumulate information on the correlation between pathogenic variants and clinical features as well as prognosis in these patients.

## HGV database

The relevant data from this Data Report are hosted at the Human Genome Variation Database at 10.6084/m9.figshare.hgv.3140, 10.6084/m9.figshare.hgv.3143, 10.6084/m9.figshare.hgv.3146, 10.6084/m9.figshare.hgv.3149, 10.6084/m9.figshare.hgv.3152.
